# Inpatient Out-of-Pocket in Iran After Health Transformation Plan

**DOI:** 10.15171/ijhpm.2018.34

**Published:** 2018-04-17

**Authors:** Sulmaz Ghahramani, Kamran Bagheri Lankarani

**Affiliations:** Health Policy Research Center, Institute of Health, Shiraz University of Medical Sciences, Shiraz, Iran.

## Dear Editor,

Piroozi et al have recently reported on out-of-pocket (OOP) payment in hospitalized patients from Sanandaj, west of Iran after health transformation plan (HTP).^1^ Their result needs to be interpreted with caution specially considering the big picture of the so called HTP. The first phase of HTP focused on hospitals affiliated to Ministry of Health and Medical Education (MoHME). MoHME aimed to reduce OOP for inpatients from 30% to 5% and 10% for insured rural and urban residents by increasing the governmental share of total health expenditure through subsidy. The aim of this plan was to protect patients against catastrophic health expenditures (CHEs) by avoiding high cash payment as OOP. The expectation was that HTP would lead to patient satisfaction after its commencement. However, a recent study showed that patients’ satisfaction was significantly reduced after the implementation of HTP.^2^ The result of this study should be interpreted with cautious because this study failed to show the overall satisfaction of health care professionals, and it seems that one-year elapse is not sufficient to assess the effect of HTP on the overall satisfaction of patients. Reports from the Iranian National Institute of Health indicated that during the first year of this plan, the overall satisfaction of patients, nurses and physicians were 30%, 24% and 22%, respectively, which seems to be mediocre.^3^

It seems that HTP is a plan in line with the goal of fifth economic, social and cultural development of Islamic Republic of Iran that had targeted households OOP reduction to less than 30% by its end.^4^ Several studies reported that although this objective was not accomplished after HTP, but there was a minor reduction in CHE since its launch.^5,6^ These studies also showed that after HTP, OOP increased, especially for out-patient services.^5^ The reason why this plan failed to significantly reduce CHE was abandoning the fact that outpatient, rehabilitative services and private sector are important drivers for OOP; nevertheless, these health expenditures were not directly targeted in HTP.^7,8^ It is worth mentioning that in recent years share of these segments in total health expenditure have increased significantly.^9^

On the contrary, Piroozi et al reported that the mean (± standard deviation, SD) of total OOP per patient admitted to hospitals affiliated to MoHME in Kurdestan province, Iran was reduced significantly after the first (*P* = .00) and third (*P* = .00) phases of the HTP from baseline from 59.4 USD, to 17.6 USD and 14.3 USD, respectively. However, there are several methodological concerns. The systematic sampling method used in this study was not adjusted for the disease severity or even diagnosis, which is not a real probability sampling method due to zero probability in some cases.^10^

In this cross-sectional analytical study, most selected wards were surgical, while the ones with prolonged hospitalization, such as oncology and medical intensive care units were not included. This could impose a bias in generalizability of their findings. In addition, obstetrics and gynecology ward with relatively high turnover was major part of their sample. As we know, during HTP, normal vaginal delivery was promoted and became free of charge in public hospitals, which might have further undermined the OOP after HTP.

In this study, in almost all cases the SD was larger than the mean estimated cost, which implies skewed distribution of the cost. In this situation the use of non-parametric comparison would have been more appropriate instead of the independent sample *t* test.^11^

Another important concern is the exchange rate of cost before and after HTP. A standard method to compare purchasing power parity of the US dollar, which was not done in this study. Instead, the authors used a fixed exchange rate of Rial to dollar for comparing the three studied phases that might have led to great discrepancy on the accuracy of this approach.

In a study in Shiraz, we compared the OOP cost of different hospital wards before and after HTP with matched controls (age, gender, and diagnosis).^8^ However, this study had smaller sample size, and was merely limited to one major hospital affiliated to Shiraz University of Medical Sciences, Shiraz, Iran. But the results revealed that although the share of OOP from total hospital cost was reduced, nevertheless, absolute OOP cost had increased significantly after HTP.

In brief, although HTP might have achieved some success in reducing the share of OOP from total hospital cost, this accomplishment is under the threat of increased absolute OOP payment, and ultimately CHE. By looking at the total health expenditure, the situation might get even worse as the OOP in private sector, both in hospitals and in out-patient services have increased, especially after raised tariffs in 2015. Further studies are suggested to justify this claim.

The timely analysis of HTP outcomes as well as other unexpected impacts, such as more workload and burn out of nurses and other healthcare staffs in university affiliated hospitals are also of importance.^12^

## Acknowledgments

The authors wish to thank Mr. H. Argasi at the Research Consultation Center (RCC) of Shiraz University of Medical Sciences, Shiraz, Iran for his invaluable assistance in editing this manuscript.

## Ethical issues

Not applicable.

## Competing interests

Authors declare that they have no competing interests.

## Authors’ contributions

SGH contributed to conception and design, drafting the article, final approval of the version, KBL contributed to interpretation of data, revising the manuscript critically for important intellectual content, final approval of the version.
